# PET-PCR method for the molecular detection of malaria parasites in a national malaria surveillance study in Haiti, 2011

**DOI:** 10.1186/1475-2875-13-462

**Published:** 2014-11-26

**Authors:** Naomi W Lucchi, Mara A Karell, Ito Journel, Eric Rogier, Ira Goldman, Dragan Ljolje, Curtis Huber, Kimberly E Mace, Samuel E Jean, Eniko E Akom, Roland Oscar, Josiane Buteau, Jacques Boncy, John W Barnwell, Venkatachalam Udhayakumar

**Affiliations:** Division of Parasitic Diseases and Malaria, Centers for Disease Control and Prevention, Center for Global Health, Malaria Branch, Atlanta, GA USA; Atlanta Research and Education Foundation, Decatur, GA USA; Laboratoire National de Santé Publique, Port au Prince, Haiti; Population Services International-Haiti, Port au Prince, Haiti; Programme National de Contrôle de la Malaria, Port au Prince, Haiti

**Keywords:** Malaria, Diagnosis, Haiti, PET-PCR

## Abstract

**Background:**

Recently, a real-time PCR assay known as photo-induced electron transfer (PET)-PCR which relies on self-quenching primers for the detection of *Plasmodium* spp. and *Plasmodium falciparum* was described. PET-PCR assay was found to be robust, and easier to use when compared to currently available real-time PCR methods. The potential of PET-PCR for molecular detection of malaria parasites in a nationwide malaria community survey in Haiti was investigated.

**Methods:**

DNA from the dried blood spots was extracted using QIAGEN methodology. All 2,989 samples were screened using the PET-PCR assay in duplicate. Samples with a cycle threshold (CT) of 40 or less were scored as positive. A subset of the total samples (534) was retested using a nested PCR assay for confirmation. In addition, these same samples were also tested using a TaqMan-based real-time PCR assay.

**Results:**

A total of 12 out of the 2,989 samples screened (0.4%) were found to be positive by PET-PCR (mean CT value of 35.7). These same samples were also found to be positive by the nested and TaqMan-based methods. The nested PCR detected an additional positive sample in a subset of 534 samples that was not detected by either PET-PCR or TaqMan-based PCR method.

**Conclusion:**

While the nested PCR was found to be slightly more sensitive than the PET-PCR, it is not ideal for high throughput screening of samples. Given the ease of use and lower cost than the nested PCR, the PET-PCR provides an alternative assay for the rapid screening of a large number of samples in laboratory settings.

## Background

The prevalence and distribution of malaria infection in a country is an integral indicator for national malaria control programmes, which can be used to measure the success of intervention strategies and for appropriate resource allocation. Therefore, an accurate and sensitive measurement of malaria parasite prevalence is crucial. Many studies have relied on the use of microscopy and rapid diagnostic tests (RDTs) for the detection of malaria parasites during epidemiological surveillance studies. The use of RDTs for malaria diagnosis increased after the World Health Organization (WHO)’s recommendation to treatment only after a parasitological diagnosis [1] RDTs are easy to use, do not require electricity or intense expertise and provide results that can be used to make treatment decisions [2]. However, neither microscopy nor RDTs are able to detect low density parasite infections [3, 4] and (reviewed in [5]) which are often asymptomatic [6–8]. Studies have demonstrated that asymptomatic malaria infections can serve as transmission foci in both low and high transmission settings and, therefore, remain as sources of new infections [9–13]. It is imperative for malaria elimination programmes to detect as many malaria parasite infections as is possible, whether symptomatic or asymptomatic [7].

Nucleic acid amplification tests (molecular tests), such as PCR-based assays, have both, superior sensitivity and specificity compared to microscopy and RDTs, detecting parasitaemia counts as low as 1 parasite/μL (reviewed in [5]). Epidemiological studies that have incorporated molecular testing show that the prevalence of malaria parasites is often underestimated when only microscopy and RDTs are utilized for parasite detection [10, 11, 14–16]. However, some challenges exist that slow/impede the implementation of molecular assays in large epidemiological surveillance studies. These include the cost and the ease of performance of molecular tests. An ideal molecular assay needs to be amenable to the screening of a large number of samples quickly and easily, as well as be cost efficient. Due to shorter assay preparation and analysis time, real-time PCR assays are better suited for large-scale studies than conventional nested 18S rRNA PCR assays. In addition, the cost of many of these assays is declining as reduced reaction volumes can be successfully utilized [17] and less expensive primer/fluorophore design utilized. One such test is the multiplexed photo-induced electron transfer (PET)-PCR which was shown to be as robust and cost-effective compared to the nested PCR [18].

Endemic malaria transmission in the Caribbean region is restricted to the island of Hispaniola: Haiti and the Dominican Republic being the two nations that share this island. A large proportion of malaria cases in Hispaniola is contributed by Haiti where 25,423 confirmed malaria cases were reported in 2012 [19]. However, accurate surveillance data from Haiti are still limited. Haiti is among the 15 countries in the Americas that are in the malaria control phase, although efforts are being mobilized to steer Hispaniola towards malaria elimination [20]. Malaria RDTs and microscopy are not always capable of detecting low parasite densities infections. Therefore, to achieve the goal of malaria elimination, more sensitive detection assays will need to be incorporated into the routine surveillance activities. To date, only one study has utilized a molecular assay to estimate the prevalence of *Plasmodium falciparum* infection in Haiti [16]. This population-based survey utilized a nested PCR assay to test a total of 714 samples obtained from the Artibonite Valley during a high malaria transmission season. Their study demonstrated higher prevalence of malaria by nested PCR (3.1%) than determined by microscopy (0.9%) [16]. Herein, the utility of the PET-PCR for use in a national community survey using samples collected in nationwide population-based surveys conducted to estimate malaria prevalence in Haiti was evaluated.

## Methods

### Ethical considerations

The survey protocol was approved by the Haiti ethical review committee. Centers for Disease Control and Prevention (CDC) investigators provided technical advice and participated without engagement, based on not having direct contact with study participants or access to any personally identifiable information.

### Survey and specimen collection

In November-December 2011, during peak malaria transmission season, a national population-based community survey was conducted by Population Services International (PSI), Haiti, in collaboration with the Haitian national malaria control programme (PNCM, per its acronym in French), and the National Public Health Laboratory (LNSP, per its acronym in French). This survey utilized a cross-sectional, two-stage, cluster design where census enumeration areas (EAs) were selected from each of Haiti’s ten departments, with a probability proportionate to size. Within each sampled EA, all households were listed, a sampling interval was determined, and 20 households were randomly selected using systematic sampling. Questionnaires were conducted with the head of the household, and all household members present on the day (total of 3,944) were tested for malaria using RDTs and microscopy; however those results will not be discussed in this manuscript. Dried blood spots (DBS) (total of 3,041) for PCR-based assays were collected on Whatman No 1 filter papers and stored at room temperature in individual bags with desiccants.

### Sample processing and DNA extraction for molecular tests

**Figure 1:**
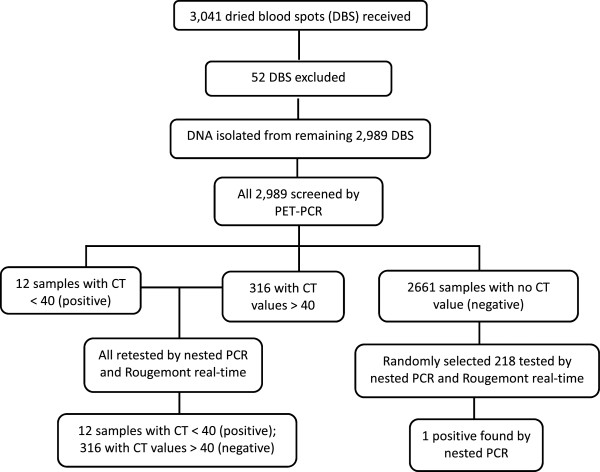
**Sample processing.**

The DBS were transferred to the CDC Atlanta, GA, USA, for molecular diagnosis. A complete database of all received DBS was established upon receipt of the samples. Each DBS was carefully examined for signs of potential contamination and to make sure there was sufficient volume of blood before DNA was extracted. Out of the 3,041 DBS received, 52 were excluded (Figure 1). DNA was successfully extracted from the remaining 2,989 samples using the QIAamp DNA Mini Kits (QIAGEN, Valencia, CA, USA) as described by the manufacturer. Briefly, three 3-mm punches of the DBS were punched out and placed into a 1.5-mL tube for processing, according to instructions. The DNA was eluted in 150 μL of elution buffer, aliquoted and stored at -20°C until use.

### PET-PCR assay

All samples were screened using the multiplex PET-PCR assay as previously described [18]. Briefly, the amplification of *Plasmodium* genus (forward primer: GGCCTAACATGGCTATGACG; reverse primer: FAM-*aggcgcatagcgcctgg*CTGCCTTCCTTAGATGTGGTAGCT) or *P. falciparum* forward primer: (ACCCCTCGCCTGGTGTTTTT and reverse primer: HEX-*aggcgcatagcgcctgg*TCGGGCCCCAAAAATAGGAA) was performed in a 20-μL reaction containing 2X TaqMan Environmental buffer 2.0 (Applied BioSystems, Grand Island, NY, USA), 125 nM each of forward and reverse primers except for the *P. falciparum* HEX-labelled primer which was used at a 62.5 nM. For each sample, duplicate PET-PCR reactions were run with 2 μL of DNA template used in the PCR reaction with the following cycling parameters: initial hot-start at 95°C for 10 min, followed by 45 cycles of denaturation at 95°C for 10 sec, annealing at 60°C for 40 sec. The correct fluorescence channel was selected for each fluorescently labelled primer-set and the cycle threshold (CT) values recorded at the end of annealing step. All assays were performed using Agilent Mx3005pro thermocyclers (Agilent technologies, Santa Clara, CA, USA).

### Validation process of the PET-PCR assay

In real-time PCR assays, the CT value is inversely proportion to the amount of DNA in a sample. Therefore, samples with high parasite densities reach the threshold earlier and have low CT values, whereas samples with low parasite densities require more cycles to reach the threshold. Conventionally, a CT value of 40 is considered as a cut-off to score a reaction as positive and, as such, a sample with a CT value above 40 is considered to be negative. In order to confirm that this cut-off for the PET-PCR assays did not miss any positive samples, samples that produced any CT value (including CT values above 40), were retested using a conventional gel-based nested PCR assay [21] and a previously described TaqMan-based real-time PCR (here after referred as Rougemont real-time PCR) [22]. In addition, a subset of PET-PCR negative samples (samples with a ‘no CT value’) were randomly selected by picking every tenth sample from the database and tested using these two methods.

### Nested 18S rRNA PCR

The nested 18S rRNA PCR assay used in this study was used as described by Singh et al. [21]. Briefly, both primary and secondary PCR reactions were performed using 2 μL DNA template in 25 μL total volume containing 1X buffer, 2.5 mM MgCl_2_, 200 μM dNTPs, 200 nM primers, and 1.25 units of Taq Polymerase (New England Biolabs, Ipswich, MA, USA). The products were analysed for the appropriate size on a 2% agarose gel with a positive being the identification of the correct base-pair size.

### Rougemont real-time PCR assay

A dual-labelled, probe-based, real-time PCR assay developed by Rougemont et al. [22] was included for comparability with the real-time PET-PCR methodology. The Rougemont real-time PCR is a duplex PCR, capable of detecting the four human-infecting *Plasmodium* species in a set of two simultaneous separate duplex reactions (*P. falciparum* duplexed with *Plasmodium vivax* and *Plasmodium malariae* duplexed with *Plasmodium ovale*). This assay was performed as described by the authors. Briefly, a 25-μL reaction containing 12.5 μL of TaqMan Universal Master Mix (Life Technologies Grand Island, NY, USA), 200 nM each of the *Plasmodium* specific forward and reverse primers and 80 nM of the species-specific probes was prepared. Two μL of template DNA was used in each reaction. The assay was executed using the following cycling conditions: an initial step at 50°C for 2 min, 95C° for 10 min, and 45 cycles of 95°C for 15 sec, and 60°C for 1 min. A cut-off CT value of 40 was used to indicate a positive result. All assays were performed using the Applied Biosystems 7500 thermocyclers (Life Technologies, Grand Island, NY, USA).

## Results

Multiplex PET-PCR was performed on all 2,989 samples (Figure 1). DNA extraction and performing the molecular tests took a dedicated team of three individuals about five months to complete. Most of the time was spent on DNA extraction procedure. Twelve samples had CT values <40 for both the genus and *P. falciparum* primers and were considered positive. A total of 316 samples had a CT value >40 by PET-PCR. These samples were retested with nested PCR and Rougemont real-time PCR and all were negative (Figure 1) by both methods. A total of 2,661 samples had a ‘no CT’ value by the PET-PCR and therefore considered negative; a subset of 218 of these negative samples were randomly selected and retested by the nested PCR and Rougemont real-time PCR. Rougemont real-time PCR produced the same results as PET-PCR, consistent with a previous demonstration showing equivalent performance [18]. However, nested PCR assay found one additional positive sample (Figure 1). The sensitivity and specificity of the PET-PCR compared to the nested PCR as a reference test was 92.3% (95% CI: 62.1-99.6%) and 100% (95% CI: 99.8-100%), respectively (Table 1).

**Table 1:** **Comparison of the PET-PCR and the nested PCR utilized in this study**

Properties	PET-PCR	Nested PCR
Detection limits (parasites/μL)	3.2	≤ 1
Species detected	Genus and *P. falciparum*	All four species
Ideal of large scale testing?	Yes	No
Cost	~$2.08	~$3.2/species
Sensitivity and specificity compared to nested PCR	92.3 and 100%	N/A

## Discussion

The CT value of 11 of the 12 positive samples was between 34 and 38, and one sample had a CT value of 28. A hundred percent concordance for these 12 samples was found when they were tested with the Rougemont real-time PCR and nested PCR. These tests also confirmed that the 12 samples were positive for *P. falciparum* and no other species of malaria parasites were detected. The mean CT value of the 12 positive samples was 35.7 giving an estimated parasitaemia of approximately 39.8 parasites/μL. This demonstrates that the average parasite density in circulation in the surveyed region is low.

In the current investigation, a malaria point prevalence of 0.4% was calculated using the PET-PCR assay. This low level of prevalence places Haiti in a good position to consider malaria elimination. Indeed, Haiti is among the 15 countries in the Americas that are in the malaria control phase and efforts are being mobilized to move towards malaria elimination [20]. The need to use more sensitive detection tools, such as molecular tests, in regions of low malaria transmission was recently proposed by a WHO Evidence Review Group [23], in March 2014. Several recommendations on the use of molecular tests in these transmission settings were put forth, including the necessity for standard operating procedures, which clearly define sample collection methods, preparation of DNA from samples and the need to use an equivalent of at least 5 μL of blood for amplification in the molecular assay. In addition, WHO’s Evidence Review Group recommended that the molecular test have a detection limit of at least 2 parasites/μL for it to be considered of “substantial improvement” over RDT and microscopy. The limits of detection of the PET-PCR were reported to be around 3.2 parasites/μL [18]. The assay, as reported, utilizes 2 μL of template DNA which was determined to be equivalent to approximately 1 μL of whole blood. Utilizing an equivalent of 5 μL whole blood, as recommended by the WHO, will most likely improve the sensitivity of the PET-PCR to approach or exceed 2 parasites/μL.

Real-time PCR assays, such as the PET-PCR, provide convenient molecular tests for the screening of large number of samples in national surveillance studies or other large-scale malaria elimination programmes such as mass screen-and-treat programmes. An important question remains: which molecular test should a programme use for success? In this study, the nested PCR detected an additional sample, most probably with parasite density below the limits of detection of the PET-PCR. However, while nested PCR assays may be slightly more sensitive than most real-time PCR assays, they are logistically not amenable to high throughput screening: the nested PCR requires two rounds of PCR amplification (resulting in its increased sensitivity) and a manual post-PCR gel electrophoresis step for visualizing results. Additionally, because the nested PCR is not a closed system, the risk of contamination can compromise tests results. Should elimination programmes be attempting to detect all ultra-low parasite densities? Clearly this will depend on the programme’s resources and the WHO’s recommendations [23] will help guide programmes on the selection of appropriate molecular tests to use in low transmission settings.

## Conclusion

This study has demonstrated the utility of the PET-PCR assay as a tool for high throughput screening for malaria parasites in a country of low malaria transmission. This technique has been transferred to Haiti National Public Health lab for use in future malaria surveys.

## References

[1] WHO (2010). Guidelines for the Treatment of Malaria. Guidelines for the Treatment of Malaria.

[2] WHO (2006). Proceedings of the WHO Informal Consultation on Development and Methods for Testing Malaria Rapid Diagnostic Tests. Towards Quality Testing of Malaria Rapid Diagnostic Tests: Evidence and Methods.

[3] Mens P, Spieker N, Omar S, Heijnen M, Schallig H, Kager PA (2007). Is molecular biology the best alternative for diagnosis of malaria to microscopy? A comparison between microscopy, antigen detection and molecular tests in rural Kenya and urban Tanzania. Trop Med Int Health.

[4] Waitumbi JN, Gerlach J, Afonina I, Anyona SB, Koros JN, Siangla J, Ankoudinova I, Singhal M, Watts K, Polhemus ME, Vermeulen NM, Mahoney W, Steele M, Domingo GJ (2011). Malaria prevalence defined by microscopy, antigen detection, DNA amplification and total nucleic acid amplification in a malaria-endemic region during the peak malaria transmission season. Trop Med Int Health.

[5] Erdman LK, Kain KC (2008). Molecular diagnostic and surveillance tools for global malaria control. Travel Med Infect Dis.

[6] Lindblade KA, Steinhardt L, Samuels A, Kachur SP, Slutsker L (2013). The silent threat: asymptomatic parasitemia and malaria transmission. Expert Rev Anti Infect Ther.

[7] mal ERACGoD, Diagnostics (2011). A research agenda for malaria eradication: diagnoses and diagnostics. PLoS Med.

[8] Bousema T, Okell L, Felger I, Drakeley C (2014). Asymptomatic malaria infections: detectability, transmissibility and public health relevance. Nat Rev Microbiol.

[9] Starzengruber P, Fuehrer HP, Ley B, Thriemer K, Swoboda P, Habler VE, Jung M, Graninger W, Khan WA, Haque R, Noedl H (2014). High prevalence of asymptomatic malaria in south-eastern Bangladesh. Malar J.

[10] Harris I, Sharrock WW, Bain LM, Gray KA, Bobogare A, Boaz L, Lilley K, Krause D, Vallely A, Johnson ML, Gatton ML, Shanks GD, Cheng Q (2010). A large proportion of asymptomatic Plasmodium infections with low and sub-microscopic parasite densities in the low transmission setting of Temotu Province. Solomon Islands: challenges for malaria diagnostics in an elimination setting. Malar J.

[11] Mosha JF, Sturrock HJ, Greenhouse B, Greenwood B, Sutherland CJ, Gadalla N, Atwal S, Drakeley C, Kibiki G, Bousema T, Chandramohan D, Gosling R (2013). Epidemiology of subpatent *Plasmodium falciparum* infection: implications for detection of hotspots with imperfect diagnostics. Malar J.

[12] Manjurano A, Okell L, Lukindo T, Reyburn H, Olomi R, Roper C, Clark TG, Joseph S, Riley EM, Drakeley C (2011). Association of sub-microscopic malaria parasite carriage with transmission intensity in north-eastern Tanzania. Malar J.

[13] Zoghi S, Mehrizi AA, Raeisi A, Haghdoost AA, Turki H, Safari R, Kahanali AA, Zakeri S (2012). Survey for asymptomatic malaria cases in low transmission settings of Iran under elimination programme. Malar J.

[14] Golassa L, Enweji N, Erko B, Aseffa A, Swedberg G (2013). Detection of a substantial number of sub-microscopic *Plasmodium falciparum* infections by polymerase chain reaction: a potential threat to malaria control and diagnosis in Ethiopia. Malar J.

[15] Steenkeste N, Rogers WO, Okell L, Jeanne I, Incardona S, Duval L, Chy S, Hewitt S, Chou M, Socheat D, Babin FX, Ariey F, Rogier C (2010). Sub-microscopic malaria cases and mixed malaria infection in a remote area of high malaria endemicity in Rattanakiri province, Cambodia: implication for malaria elimination. Malar J.

[16] Eisele TP, Keating J, Bennett A, Londono B, Johnson D, Lafontant C, Krogstad DJ (2007). Prevalence of *Plasmodium falciparum* infection in rainy season, Artibonite Valley, Haiti, 2006. Emerg Infect Dis.

[17] Kamau E, Alemayehu S, Feghali KC, Saunders D, Ockenhouse CF (2013). Multiplex qPCR for detection and absolute quantification of malaria. PLoS One.

[18] Lucchi NW, Narayanan J, Karell MA, Xayavong M, Kariuki S, DaSilva AJ, Hill V, Udhayakumar V (2013). Molecular diagnosis of malaria by photo-induced electron transfer fluorogenic primers: PET-PCR. PLoS One.

[19] Pan American Health Organization (2013). Situation of Malaria in the Region of the Americas, 2000-2012.

[20] Roberts L (2010). Elimination meets reality in Hispaniola. Science.

[21] Singh B, Bobogare A, Cox-Singh J, Snounou G, Abdullah MS, Rahman HA (1999). A genus- and species-specific nested polymerase chain reaction malaria detection assay for epidemiologic studies. Am J Trop Med Hyg.

[22] Rougemont M, Van Saanen M, Sahli R, Hinrikson HP, Bille J, Jaton K (2004). Detection of four Plasmodium species in blood from humans by 18S rRNA gene subunit-based and species-specific real-time PCR assays. J Clin Microbiol.

[23] WHO Evidence Review Group on Malaria Diagnosis in Low Transmission Settings. http://www.who.int/malaria/mpac/mpac_mar2014_diagnosis_low_transmission_settings_report.pdf]

